# Synbiotics for Broiler Chickens—*In Vitro* Design and Evaluation of the Influence on Host and Selected Microbiota Populations following *In Ovo* Delivery

**DOI:** 10.1371/journal.pone.0168587

**Published:** 2017-01-03

**Authors:** Aleksandra Dunislawska, Anna Slawinska, Katarzyna Stadnicka, Marek Bednarczyk, Piotr Gulewicz, Damian Jozefiak, Maria Siwek

**Affiliations:** 1 Department of Animal Biochemistry and Biotechnology, UTP University of Science and Technology, Bydgoszcz, Poland; 2 Poznań Science and Technology Park of the AMU Foundation, Poznan, Poland; 3 Department of Animal Nutrition and Feed Management, Poznan University of Life Sciences, Poznan, Poland; University of Illinois at Urbana-Champaign, UNITED STATES

## Abstract

Synbiotics are synergistic combinations of prebiotics and probiotics. In chickens, synbiotics can be delivered *in ovo* to expedite colonization of the gut by beneficial bacteria. We therefore aimed to design synbiotics *in vitro* and validate them in broiler chickens upon *in ovo* delivery. The probiotic components of the synbiotics were *Lactobacillus salivarius* and *Lactobacillus plantarum*. Their growth was assessed in MRS medium supplemented with different prebiotics. Based on *in vitro* results (hatchability and growth curve), two synbiotics were designed: S1 –*Lactobacillus salivarius* with galactooligosaccarides (GOS) and S2 –*Lactobacillus plantarum* with raffinose family oligosaccharides (RFO). These synbiotics were delivered to Cobb broiler chicken embryos on day 12 of incubation at optimized doses (10^5^ cfu egg^-1^ of probiotic, 2 mg egg^-1^ of prebiotic). Post hatching, 2,400 roosters were reared (600 individuals group^-1^ divided into eight replicate pens). Microbial communities were analyzed in ileal and cecal digesta on day 21 using FISH. Gene expression analysis (*IL1β*, *IL4*, *IL6*, *IL8*, *IL12*, *IL18*, *IFNβ*, and *IFNγ*) was performed on days 7, 14, 21, and 42 for the spleen and cecal tonsils with RT-qPCR. Body weight and feed intake of the roosters did not differ by the treatments. Microbial populations of *Lactobacillus* spp. and *Enterococcus* spp. in the ileum were higher in S1 and S2 than in the control. In the cecum, the control had the highest bacterial counts. S1 caused significant up-regulation of *IL6*, *IL18*, *IL1β*, *IFNγ*, and *IFNβ* in the spleen on day 21 and *IL1β* on day 7 (*P* < 0.05). In cecal tonsils, S1 caused significant down-regulation of *IL12*, *IL8*, and *IL1β* on day 42 and *IFNβ* on day 14 (*P* < 0.05). S2 did not elicit such patterns in any tissues investigated. Thus, we demonstrate that divergent effects of synbiotics in broiler chickens were reflected in *in vitro* tests.

## Introduction

Microbiota of the avian gastrointestinal tract (GIT) constitute a key factor in the development and regulation of immunity, digestion, and absorption of nutrients and their metabolism [1,2]. GIT microbiota can be modulated by bioactive substances, such as prebiotics, probiotics, or synbiotics [3]. These bioactive compounds can directly modulate the host microbiome and, consequently, indirectly affect host organisms. The term “synbiotic” is used to describe synergistic combinations of prebiotics and probiotics [4]. Two types of synergism between prebiotic and probiotic have been defined. Both compounds can be synergistic with each other, or alternatively with the host [5,6]. In the first case, the prebiotic stimulates growth of probiotic bacteria. The second mechanism assumes that the prebiotic and probiotic act independently in the GIT where they stimulate development of the host microbiota. Indigestible oligosaccharides (prebiotics) are fermented in the GIT, while beneficial live microorganisms (probiotics) colonize the GIT [7].

The largest group of microorganisms classified as probiotics are the lactic acid bacteria (LAB). They prevent pathogens from attaching and proliferating in the intestinal mucosa. LAB are also release enzymes into the intestinal lumen [8]. Lactobacilli are prevalent members of the GIT microflora in various livestock species [9]. To be used as probiotics, these bacteria must be able to survive in the intestine and exhibit antagonistic properties against harmful bacteria. Therefore, the most important feature of probiotic strains is their ability to adhere to intestinal epithelial cells [10]. Prebiotics, such as inulin, galactooligosaccharides (GOS), fructooligosaccharides (FOS), mannan-oligosaccharides (MOS), and the raffinose-family oligosaccharides (RFO), are used to improve and maintain optimal intestinal functionality by stimulating the growth and biodiversity of beneficial microbiota and reducing the proliferation of pathogenic strains [11]. A single dose of prebiotic increases the level of beneficial bacteria in the intestinal tract of adult organisms [12]. Both prebiotics and synbiotics have a positive effect on gut-associated lymphoid tissue (GALT). It was demonstrated that both substances administered *in ovo* stimulated the development of GALT after hatching. Synbiotics have a strong stimulating effect on the colonization of GALT by T and B cells [13]. It is also documented that in chickens, as well as in other animals, the effectiveness of prebiotics increases when they are used as part of a synbiotic [14].

Effective modulation of the GIT microbiota depends on the method and timing of bioactive compound delivery [3]. Routinely, prebiotics, probiotics, or synbiotics are provided in-feed or in-water immediately after hatching [15]. The effectiveness of early post-hatching supplementation with bioactive compounds is high because this is the period (from hatching to the second week) when the GIT is first colonized by microbiota and when GALT becomes functionally mature [16,17]. Alternatively, prebiotics, probiotics, or synbiotics can be delivered *in ovo* into the chicken embryo, which extends the effective time of action to the pre-hatching period. The *in ovo* method of delivery of microflora-promoting bioactive compounds, which was developed by our group, is based on a single dose of prebiotics or synbiotics injected into the air cell precisely on day 12 of egg incubation [18–20]. Downstream effects of *in ovo* injection depend on proper timing of administration as well as the type and dose of the bioactive compound; therefore, it is crucial to screen the biological properties of these compounds and validate their effectiveness for *in ovo* delivery [11,12,14,15].

Effects of *in ovo* delivery on microflora-promoting bioactive compounds are detectable during the entire lifespan of the chicken [18,21]. In our previous experiments, we demonstrated that a single injection of prebiotic or synbiotic *in ovo* could modify the entire spectrum of phenotypic traits in growing broiler chickens, including growth traits [22], immune organ structure and development [13,18,21], histological composition of the intestinal tissue [23,24], parameters of meat quality [25], digestive potency of the pancreas [26], and molecular changes in the cecal tonsils, spleen [17,18], and liver (A. Dunislawska, personal communication). However, the reported influence of *in ovo* treatment on the animal was strongly dependent on properties of the given prebiotic (e.g., inulin, GOS, RFO, or beta-glucans). These effects are more complex for synbiotics containing at least two biologically active compounds, which exert different effects on the organism when they are delivered alone than when they are delivered together. Based on numerous studies, careful pre-selection of the synbiotic and its validation for animal studies is crucial. Because *in vivo* examination of bioactive properties of prebiotics, probiotics, and synbiotics is time-consuming, labor-intensive, and require large numbers of animals, *in vitro* assays should be preferentially used [27].

In this study, we hypothesized that *in vitro* screening of synbiotics prior to *in vivo* study could be indicative of the biological properties synbiotics and their impact on the host. The goal of this study was to design *Lactobacillus* synbiotics based on *in vitro* testing and validate their impact on broiler chickens after *in ovo* injection into 12-day-old embryos. In our experiment, we assessed the impact of treatment with specific synbiotics on the microbiome and host. The animals were studied from hatching to market age under commercial conditions using performance and molecular responses as indicators of the effectiveness of the treatments.

## Materials and Methods

### Testing trial

#### Lactobacilli synbiotics selection *in vitro*

For *in vitro* trials, two *Lactobacillus* strains were chosen, based on their proven probiotic activities in chickens: *Lactobacillus (L*.*) salivarius* IBB3154 and *L*. *plantarum* IBB3036. Both strains were derived from the collection of the Institute of Biochemistry and Biophysics (IBB), Polish Academy of Sciences (PAS) in Warsaw, Poland. For synbiotic design, several prebiotics were tested: inulin (Sigma-Aldrich GmbH, Schnelldorf, Germany), RFO [28], GOS (trade name: Bi^2^tos; Clasado Biosciences, Ltd., Jersey, UK), and beta-glucans (trade name: LactoShield; BioAtlantis, Ltd., Tralee, Ireland). Growth curves of these bacteria in the presence of different prebiotics were estimated using an automated growth analyzer Bioscreen C (Oy Growth Curves Ab Ltd). The total volume of the well in the Bioscreen plate was 400 μL and was filled with a test solution comprising 360 μL of MRS medium [29] and 40 μL of inoculum of each *Lactobacillus* bacteria. Several variants of MRS medium were used. These consisted of a standard with glucose and one of four modified MRS media in which glucose was replaced with inulin, RFO, GOS, or beta-glucans [30,31]. Inocula were prepared as follows: tested strains (*L*. *salivarius* IBB3154 and *L*. *plantarum* IBB3036) were inoculated in tubes with standard MRS medium with glucose and incubated for 16 h at 37°C [32,33]. Next, they were centrifuged (4000× *g* for 15 min) and suspended in peptone water. Suspensions were mixed thoroughly and diluted to obtain OD 1 at 600 nm corresponding to 10^6^ cfu cm^-3^. Growth of the tested strains, *L*. *salivarius* IBB3154 and *L*. *plantarum* IBB3036, was measured turbidimetrically hourly for 72 h using a wide-range filter (420 nm–580 nm) with 10 s mixing before each measurement. All measurements were performed in triplicate.

#### Doses optimization *in vivo*

To evaluate doses of synbiotics for the *in vivo* study, trials were performed in which effects of synbiotics delivered *in ovo* on egg hatchability was determined for *L*. *salivarius* IBB3154 with GOS (S1) and *L*. *plantarum* IBB3036 combined with RFO (S2). A sample of 4,200 Cobb500FF eggs was randomly distributed into 14 experimental groups. On day 12 of embryo development, a control group was mock injected *in ovo* with 0.2 mM physiological saline (0.9%). Another group was used as a negative control without *in ovo* injection. The remaining 12 experimental groups were used for synbiotic injection *in ovo* with six different doses of each synbiotic. Doses were prepared by combining the three levels of bacterial counts (10^3^, 10^4^, 10^5^ bacteria cfu egg^-1^) with two levels of prebiotic concentrations (2 or 5 mg egg^-1^). The experimental setup is presented in the Tab A in S1 File. Application of 0.2 mL of aqueous solution into the air cell of each egg was performed with a syringe and 4-mm-long needle. After injection, the hole was sealed with non-toxic glue to avoid embryo contamination and prevent moisture loss. After *in ovo* injection, eggs were further incubated until hatching. The analysis of hatchability was performed in a commercial hatchery (DrobexAgro, Solec Kujawski, Poland). Statistical analyses of hatchability were conducted using a one-way analysis of variance (ANOVA) and Duncan’s post hoc test to compare means between experimental groups.

### Field trial

#### *In ovo* injection and tissue collection

For the field trial with optimized doses of synbiotics, 5850 Cobb500FF eggs were incubated in a commercial hatchery using an automated incubator at 37.8°C and a relative humidity of 61%–63%. Egg weight was approximately 65 grams. Eggs used for experiment originated from a herd at the age of 42 weeks. On day 12 of incubation, eggs were randomly distributed into two experimental groups: S1, S2 and the control group. Eggs were injected *in ovo* with 0.2 mL of either S1 or S2 aqueous solution. Synbiotics consisted of 10^5^ bacteria cfu egg^-1^ and 2 mg egg^-1^ of prebiotic. The control group (C) was mock-injected with 0.2 mM physiological saline (0.9%). Additionally, an uninjected control (U) was incubated in parallel. Eggs were incubated until hatching. At hatching, hatchability was scored to determine mortality of the embryos resulting from *in ovo* injection. A total of 2,400 roosters was moved to a commercial farm (Piast, Olszowa Experimental Unit 0161, Poland) for rearing. Animals from each experimental group (600 individuals group^-1^) were split into 8 pens (75 individuals pen^-1^). Each pen was considered a replicate.

During the entire rearing period, chickens had free access to feed and fresh drinking water. Mortality, body weight, fodder consumption, and feed conversion efficiency (FCE) were recorded on a regular basis. FCE was determined using residual feed intake (RFI) and residual growth (RIG). RFI was defined as the difference between observed and predicted feed intake. On days 7, 14, 21, and 42 post hatching, five randomly selected individuals (one from each experimental group) were euthanized. Tissues (spleen, cecal tonsils, and jejunum) were collected and stabilized in *fix* RNA solution (EURx, Gdansk, Poland). Overall, 60 broiler chickens (4 collection times × 3 groups × 5 individuals per group) were used for gene expression analysis. The remaining 1,980 roosters were used to estimate production parameters. For the analyses of gastrointestinal microbial communities, the contents of the ileum and ceca collected from two birds per pen were pooled (eight replicates of approximately 10 g) on 21 day of life. Samples were immediately frozen and stored at -80°C for the microbiota composition analysis by fluorescent *in situ* hybridization of single bacterial cells (FISH).

During the entire rearing period, all experimental broilers were under veterinary care. Chickens exhibiting any signs of severe illness or moribundity were euthanized. Animal use was approved by the Local Ethical Committee for Animal Experimentation, University of Sciences and Technology, Bydgoszcz, Poland 36/2012 on July 12, 2012.

#### Microbial community analysis by fluorescent in situ hybridization (FISH)

From all treatments 2 birds from 9 pens was sacrificed (n = 18) by cervical dislocation. The samples of the digesta from different gastrointestinal segments (ileum, caeca) were collected and randomly pooled into six replicates per treatment, three birds per sample. For FISH analysis, 100 μL of the ileal digesta was diluted in PBS and pipetted onto 0.22 μm polycarbonate filters (Frisenette, Knebel, Denmark) and vacuumed with a vacuum pump (KNF Neuberger GmbH, Freiburg, Germany). After vacuuming, filters were transferred onto cellulose discs for dehydration in growing dilutions of ethanol (50, 80, and 96%, 3 min each). For each sample, a series of identical filters was prepared to determine optimal hybridization. Oligonucleotide probes used for this study (Tab B in S1 File) were selected based on the results of previous studies [69–72]. Hybridizations were conducted in 50 μL of hybridization buffer (0.9 M NaCl; 20 mM Tris/HCl, pH 7.2; 0.01% SDS) containing the oligonucleotides probes. After hybridization, filters were washed with a washing buffer (20 mM Tris/HCl, pH 7.2; 0.01% SDS; 5 mM EDTA) for 20 min at 48°C. After washing, filters were rinsed gently in distilled water, air-dried, and mounted on slides with VectaShield (Vector Laboratories Inc., Burlingame, CA, US), an anti-fading agent containing DAPI (4',6-diamidino-2-phenylindole). To distinguish bacteria from other particles in the ileal samples (DAPI), filters were left at 4°C for 1 h in the dark until visualization with a Carl Zeiss Microscope Axio Imager M2 (Carl Zeiss Jena GmbH, Jena, Germany), as described elsewhere [34,35]. A detailed FISH protocol is presented in the Fig A in S1 File. From one replicate (polycarbonated filter) 50 areas were measured and used as means in calculation. Statistical analyses of the selected microbiota populations represent 6 pooled replicates from 18 birds per group; each visualised by fluorescent microscopy 50 times.

#### RNA isolation and reverse transcription quantitative PCR (RT-qPCR)

Prior to total RNA isolation, tissues were homogenized with the TissueRuptor homogenizer (Qiagen GmbH, Hilden, Germany) in TRIzol® LS Reagent (Ambion/Thermo Fisher Scientific, Valtham, USA). Further steps of isolation were performed with a commercial kit (Universal RNA Purification Kit, EURx, Gdansk, Poland). RNA quality and quantity was controlled by electrophoresis on 2% agarose gel and NanoDrop 2000 (Scientific Nanodrop Products, Wilmington, USA). In addition, 10% of samples were used to control the integrity of RNA on automated electrophoresis with the Experion Automated Electrophoresis System (BioRad, Hercules, California, USA).

cDNA was synthesized using Maxima First Strand cDNA Synthesis Kit for RT-qPCR (Thermo Scientific/Fermentas, Vilnius, Lithuania), following the manufacturer’s recommendations. Obtained cDNA was diluted to 70 ng μl^-1^ and stored at -20°C. RT-qPCR reactions were conducted with a total volume of 10 μL. The reaction mixture included Maxima SYBR Green qPCR Master Mix (Thermo Scientific/Fermentas, Vilnius, Lithuania), 1 μM of each primer, and 2 μl of diluted cDNA (140 ng). Thermal cycling was performed in a LightCycler II 480 (Roche Diagnostics, Basel, Switzerland). Each RT-qPCR reaction was conducted in two technical replicates. Gene expression analysis was performed for the selected panel of cytokines: Th1 (*IFNγ*, *IL12*), Th2 (*IL4*), proinflammatory (*IL6*, *IL18*, and *IL1β*), antiviral (*IFNβ*), and chemokine (*IL8*). Sequences of primers were based on the literature [19,73–76] or designed based on cDNA nucleotide sequence using NCBI Primer Blast [36] (Tab C in S1 File).

#### Relative quantification of gene expression and statistical analysis

Relative gene expression analysis was conducted separately for each experimental group by the ΔΔCt method [37] using *UB* and *G6PD* as reference genes. Geometric means of Ct (cycle threshold) values of reference genes were used in the analysis. For each of the samples, Ct differences between target and reference genes were calculated. Control (C) samples were used as calibrators. ΔΔCt was calculated by deducting the ΔCt value of the calibrator from ΔCt of the unknown sample. For the calculation of the normalized expression level of the gene, the following formula was used: R = 2 –^ΔΔCt^ [37]. Statistical analyses were performed by comparing the Ct value of each experimental group with that of the control group by Student's *t*-test (*P* < 0.05). Standard errors of the mean (SEM) were applied as a parameter of variation within the groups.

## Results

### Lactobacilli–synbiotic selection *in vitro*

**Fig 1:**
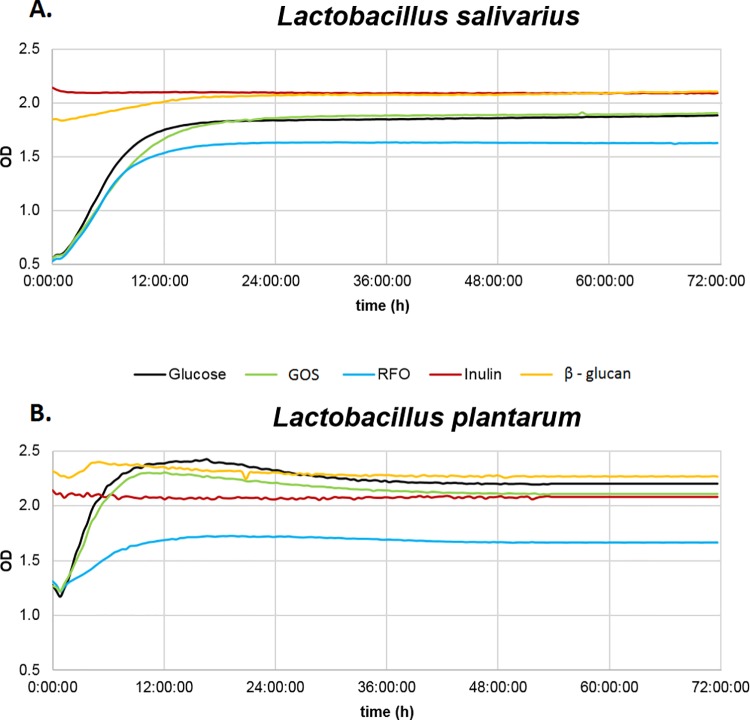
Changes in optical density (OD) of culture media during incubation of *Lactobacillus* bacteria with prebiotics. Incubation was performed with glucose as a control for (A) *Lactobacillus salivarius* and (B) *Lactobacillus plantarum* in presence of prebiotics RFO, Inulin, GOS, or beta-glucan. Growth of bacteria in the MRS medium supplemented with the prebiotics glucose, GOS, RFO, inulin, or beta-glucan was measured using the automated growth analyzer Bioscreen C (Oy Growth Curves Ab, Ltd.) for 72 h.

Results of turbidimetric measurements of tested bacteria in the presence of five prebiotics are presented in Fig 1A (*L*. *salivarius*) and Fig 1B (*L*. *plantarum*). For *L*. *salivarius*, the optimal growth curve was observed in the presence of GOS and the MRS standard medium with glucose. Bacterial growth in the standard MRS medium supplemented with RFO was much weaker than that in MRS medium with glucose. Maximum optical density was achieved in *L*. *salivarius* cultures with GOS and was 0.06 OD higher than in the MRS culture with glucose. The optimal growth curve of *L*. *plantarum* was detected for the MRS standard medium with glucose. Comparably high growth curves of *L*. *plantarum* were observed in the presence of GOS and RFO in the MRS medium. Growth of *L*. *plantarum* in the MRS standard medium with glucose was 0.74 OD higher than that in MRS medium with RFO. Bacterial growth in the MRS standard medium supplemented with either inulin or beta-glucans was much weaker than that in the MRS standard medium with glucose.

### Dose optimization in testing trial

**Fig 2:**
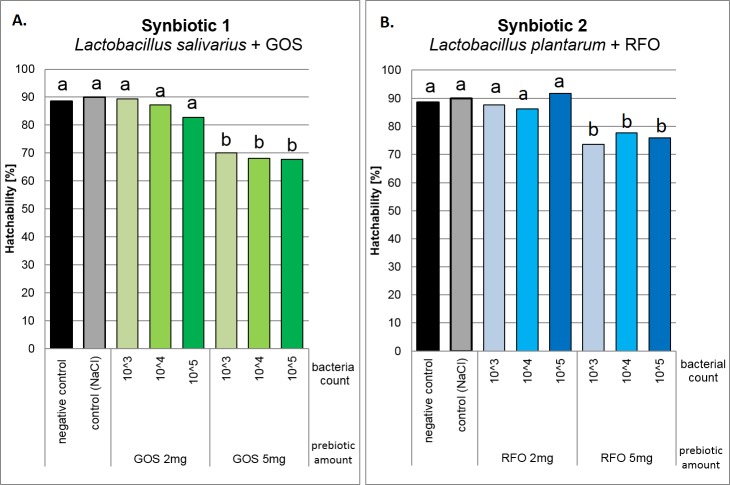
**Chick hatchability in response to different doses of synbiotics delivered *in ovo* for (A) *L*. *salivarius* + GOS (S1) and (B) *L*. *plantarum* + RFO (S2).** Six doses of two synbiotics were delivered at 12^th^ day of incubation. Combination of three doses of probiotics (10^3^, 10^4^, 10^5^ cfu egg^-1^) with two doses of prebiotics (2 mg egg^-1^ or 5 mg egg^-1^) were tested. The highest dose that did not decrease hatchability (10^5^ cfu egg^-1^ of prebiotic and 2 mg egg of probiotic) was selected for the field trial. One-way analysis of variance (ANOVA) was performed. Duncan’s post hoc test was applied to compare the mean values between pairs in the experimental groups (a, b: P < 0.05).

Significant differences in hatchability of eggs injected *in ovo* were observed between two doses of prebiotics, 2 mg egg^-1^ and 5 mg egg^-1^. In this experiment 4,200 eggs were distributed to 14 groups. Hatchability was significantly higher after administration of 2 mg egg^-1^ GOS (Fig 2A) or RFO (Fig 2B). Higher prebiotic concentration (5 mg) caused significant decline in egg hatchability. A hatchability of approximately 90% was observed after S1 injection containing 10^5^ of *L*. *salivarius* and 2 mg of GOS and after S2 injection of 10^3^ or 10^5^ of *L*. *plantarum* and 2 mg of RFO. Hatchability in the control group (C) was 90%, whereas in the uninjected (U) group was 89%. Based on these results, the optimized dose of both S1 and S2 synbiotics was 2 mg of prebiotic and 10^5^ of bacteria. The aim of the selection process was to determine the highest level of bioactive compound that did not impede hatchability.

### Broiler chicken performance in the field trial

For the field trial, based on 5,850 eggs, hatchability was 89.1%, 91.6%, and 91.9% in the S1, S2, and C groups, respectively. Body weight differed only on the first day after hatching. Chickens from the S2 group (41.6 g) were significantly heavier than those in the S1 (40.3 g) and C (40.7 g) groups (*P* < 0.05). We did not find any differences in body weight gain (BWG) among the groups over the entire rearing period. The lowest feed intake (FI) between day 1 and 10 was observed in the S1 group (247 g) and differed significantly from the C (254 g) and S2 (258 g) groups. For the entire rearing period, FI in the S1 group (4,930 g) was comparable to the C (4,940 g) group. The lowest feed consumption (4,898 g) was observed in the S2 group. Feed conversion efficiency (FCE) calculated for the entire rearing period (day 1 to 42) was almost identical for all groups and equal to 1.60 g g^-1^ in the synbiotic-treated groups and 1.59 g g^-1^ in the C group. Mortality was numerically lower in the synbiotic-treated groups and equal to 0.83% (S1) and 1.17% (S2). Numerically, the highest chicken mortality was detected in the C group (1.83%). Chicks performance (BWG, FI, FCE and mortality) carried out on 1,980 broilers to estimate production parameters in response to different synbiotics delivered *in ovo* is presented in the Tab D in S1 File.

### Microbial community analysis by fluorescent in situ hybridization (FISH)

**Fig 3:**
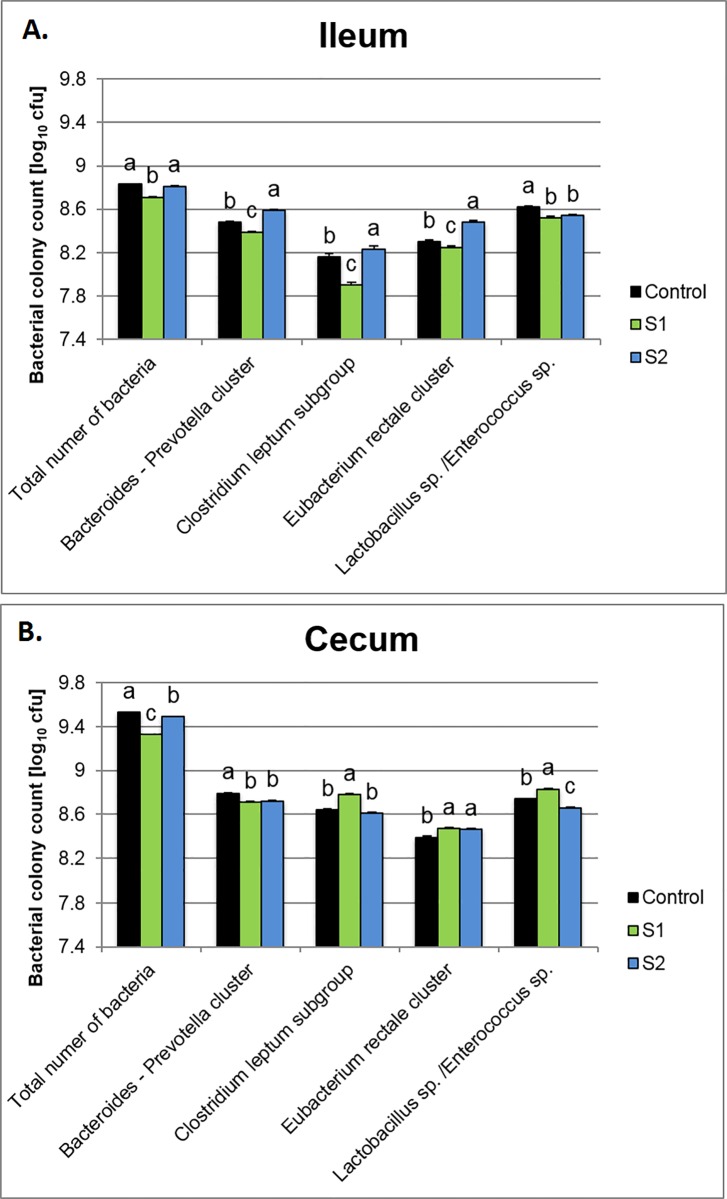
Selected bacterial counts (log cfu/ml digesta) in digesta determined by DAPI staining (total number of bacteria) and fluorescent in situ hybridization (FISH). Hybridization was performed with oligonucleotides, whose sequence was based on the literature. A Carl Zeiss Microscope Axio Imager M2 (Carl Zeiss Jena GmbH, Jena, Germany) was used to determine bacterial count.

In the ileal digesta, irrespective of the treatment, application of the synbiotics lowered *Lactobacillus* sp.*/Enterococcus* sp. Other microbiota populations exhibited different responses. S1 (Fig 3A) decreased the total number of bacteria, as well as the *Bacteroides-*Prevotella cluster, *Clostridium leptum subgroup*, and the *Eubacterium rectale* cluster (P = 0.0001). In contrast, in the S2 group, higher counts of the *Bacteroides-*Prevotella cluster, *Clostridium leptum subgroup*, and the *Eubacterium rectale* cluster (P = 0.001) were determined. In cecal digesta (Fig 3B), the highest number of total bacteria was detected in the C group (P = 0.0001). Both synbiotics lowered the *Bacteroides-*Prevotella cluster and increased the *Eubacterium rectale* cluster. Moreover, in the S1 group, higher counts of *Lactobacillus* sp. */Enterococcus* sp. were observed (P = 0.0001).

### Immune-related gene expression signatures

**Fig 4:**
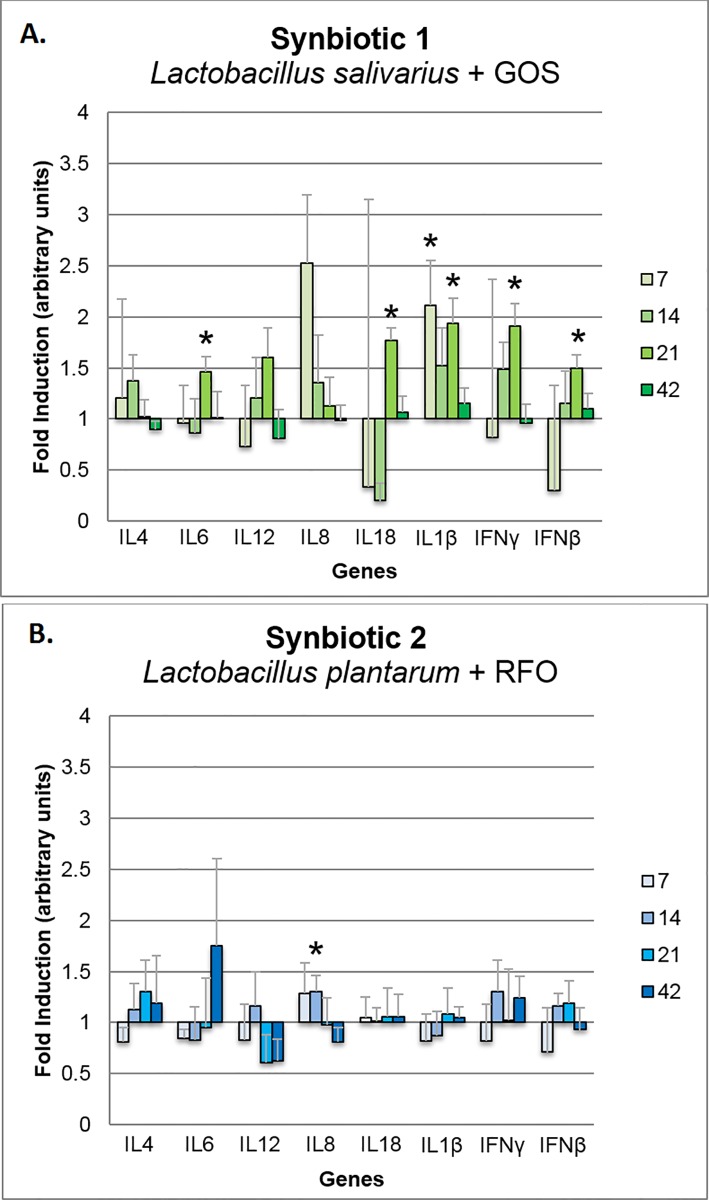
**Changes in relative expression of the immune-related panel of genes in the spleen of broiler chickens injected *in ovo* with the synbiotics (A) *Lactobacillus salivarius* with GOS and (B) *Lactobacillus plantarum* with RFO.** Analysis was performed using the ^ΔΔ^Ct method to determine fold induction. Synbiotics were injected on day 12 of embryonic development. Sampling days were 7, 14, 21, and 42 days post hatching. Statistical analysis consisted of comparing the experimental groups with the control group by Student's *t*-test; * for P < 0.05. Each bar represents the mean (*n* = 5) ± standard error of the mean (SEM).

Based on biological material collected from 60 broilers for expression analysis we can infer that *in ovo* stimulation with S1 caused significant up-regulation of *IL6*, *IL18*, *IL1β*, *IFNγ*, and *IFNβ* in the spleen on day 21 and the *IL1β* on day 7 (*P* < 0.05) (Fig 4A). S1 stimulation induced a pattern of up-regulation of gene expression in the spleen for the panel of genes at the four time points: 7, 14, 21, and 42 days of life. Administration of S2 did not cause any significant pattern of immune-related gene modulation in the spleen (Fig 4B). Significant up-regulation was detected for *IL8* on day 14 and down-regulation for *IL12* on day 42 (*P* < 0.05). Analysis was performed based on 5 biological repeats, and every in 2 technical repeats.

**Fig 5:**
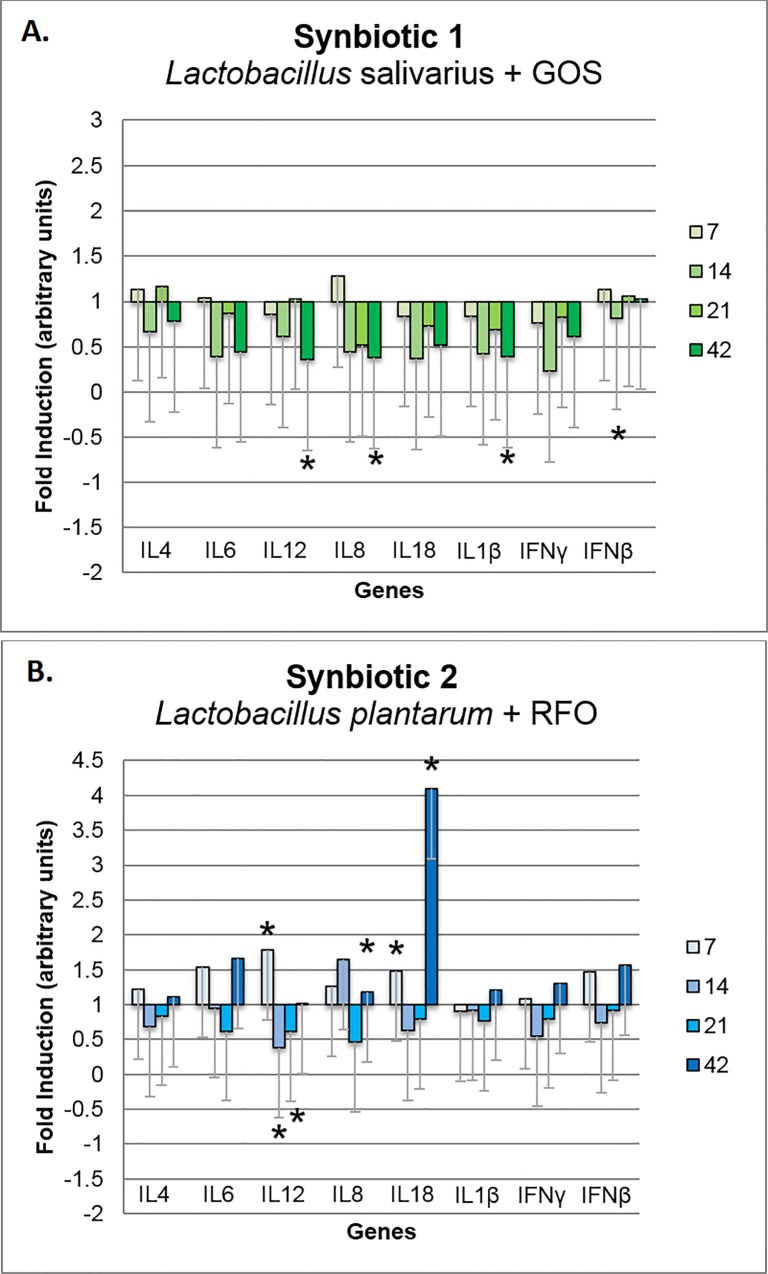
**Changes in relative expression of immune-related panel of genes in the cecal tonsils of broiler chickens injected *in ovo* with synbiotics (A) *Lactobacillus salivarius* with GOS and (B) *Lactobacillus plantarum* with RFO.** Analysis was performed using the ^ΔΔ^Ct method to determine fold induction. Synbiotics were injected on day 12 of embryonic development. Sampling days were 7, 14, 21, and 42 days post hatching. Statistical analysis consisted of comparing the experimental groups with the control group by Student's *t*-test; * for P < 0.05. Each bar represents the mean (*n* = 5) ± standard error of the mean (SEM).

In the cecal tonsils, S1 caused a significant down-regulation of *IL12*, *IL8*, and *IL1β* on day 42 and *IFNβ* on day 14 (*P* < 0.05) (Fig 5A). In this tissue, there was a clear down-regulatory pattern of the gene expression induced by the S1 treatment. Conversely, S2 did not cause a pattern of immune-related gene expression in the cecal tonsils. Up-regulation of gene expression was detected on day 7 for the genes *IL12* and *IL18* and on day 42 for genes *IL8* and *IL18*. *IL12* was down-regulated on days 14 and 21 (*P* < 0.05) (Fig 5B). Immune-related gene expression in the jejunum in the S1 and S2 groups did not differ significantly from that in the C group.

## Discussion

### Lactobacilli–synbiotic selection *in vitro*

During *in vitro* selection of different compositions of lactobacilli and prebiotics, growth curves were estimated to determine potency of the given LAB strain to metabolize prebiotic oligosaccharides. Growth of the two tested LAB strains in the presence of four prebiotics was compared to the reference medium, standard MRS medium with glucose. Bacterial growth in the reference medium was defined as having the optimal logarithmic growth curve, and as such, it was treated as the reference growth curve for further comparisons. In our analysis, the logarithmic growth curve was constructed for both LAB strains (*L*. *salivarius* and *L*. *plantarum*) in the presence of GOS and RFO. The growth curve of *L*. *salivarius* in the MRS medium with GOS exhibited a comparable slope and height to that of growth curve in the MRS medium with glucose. This confirmed that *L*. *salivarius* utilizes GOS as effectively as glucose. Conversely, growth of *L*. *plantarum* in the MRS medium with RFO was lower than that in the MRS medium with glucose, although the slope of the curve was similar. This could be explained by the more complex structure of the saccharides present in RFO (i.e., trisaccharide-raffinose, tetrasaccharide-stachyose, and pentasaccharide-verbascose [22,28]. These results are in accordance with findings of Gulewicz et al. [31], who reported that growth of LAB in MRS medium with RFO was less potent than in that in MRS medium with glucose. There are other reports that confirm the variation in utilization of prebiotics by bacteria, which largely depends on the LAB strain [38,39]. It could be concluded that *L*. *salivarius* metabolization of GOS is almost as effective as a basic source of carbon (i.e., glucose), but the *L*. *plantarum* RFO prebiotic provided a less efficiently metabolizable source of carbon.

The best combination of prebiotic and probiotic (i.e., synbiotic) for *in ovo* injection can be seen from two different perspectives. First, the most suitable prebiotic for the probiotic bacteria injected *in ovo* is the one that supports growth of the probiotic component. In this case, an optimally combined synbiotic allows for more efficient use of both components because the viability of the probiotic bacteria is improved by using the prebiotic as a substrate for fermentation [40]. We define such a synbiotic as exerting synergistic effects towards the probiotic. Second, if the prebiotic is less efficiently used by the probiotic bacteria, it remains available to other indigenous stains of intestinal microbiota. In this situation, the prebiotic has a positive influence on the host organism through improvement of microbial balance in the intestines [41]. In our opinion, such synbiotic can be defined as synergistic toward the host. In the next steps of the *in vivo* experiments, two synbiotics (i.e., *L*. *salivarius* + GOS and *L*. *plantarum* + RFO) were designed based on *in vitro* results. These synbiotics were expected to present the two types of synergism explained above (i.e., synergism between the prebiotic and probiotic components [S1] and synergism between the two independent bioactive compounds and the host [S2].

### Dose optimization *in vivo*

Use of *in ovo* technology for delivering microbiota-promoting bioactive compounds in chickens has several advantages over in-feed supplementation, including uniformity and precision of bioactive compound delivery to each embryo, low usage of those compounds, optimal timing for stimulating gut colonization with beneficial bacteria, as well as encouraging maturation of the immune system. The associated risks, however, includes potential impediment of embryo viability and hatchability because of improper injection technique or dose of the bioactive compounds. In our case, the injection technique had been optimized and, in every study conducted to date, including the current one, the hatchability score was over 90%. The injection site is an air cell within the egg and the timing, was at day 12 of incubation. This approach is least invasive for the embryo and allows for easy automatization without compromising egg hatchability. The other component that influences hatchability (i.e., doses of bioactive compounds for *in ovo* injection) must be optimized prior to field trials. In our studies, we assumed the criterion of hatchability as the major indicator of the optimal dosage [42]. By definition, we selected the highest dose of a given bioactive compound that did not cause significant reduction in hatchability. In our case, increasing bacterial count in the synbiotic did not influence the hatchability score. However, there was a considerable decrease in hatchability of the eggs that had been injected *in ovo* with synbiotics containing 2 mg as against those injected with 5 mg of GOS or RFO prebiotic. Such a difference can be explained by overstimulation of the bacterial population in the embryonic gut, which interfered with embryo development.

### Broiler chicken performance in the field trial

In the current experiment, synbiotics composed of *Lactobacillus* LAB species combined with GOS (S1) or RFO (S2) injected *in ovo* did not influence final BWG and FCE. In the previous trials, we observed changes in broiler performance traits (BWG and FCE) introduced by *in ovo* treatment with prebiotics and synbiotics. For example, *in ovo* delivery of two synbiotics composed of inulin + *Lactococcus lactis* subsp. *lactis* or GOS + *Lactococcus lactis* subsp. *cremoris*, significantly increased final BWG with unchanged FCE [21]. In another experiment, *in ovo* injection of GOS and RFO prebiotics alone also increased BWG, but with higher FI and FCE [42]. Changes in BWG and FCE in animals supplemented (*in ovo* or in-feed) is quite natural and it results from competition for nutrients between the host and its intestinal microbiota, which usually results in higher energy uptake [43]. The lack of changes in performance traits observed in this study was considered beneficial, given that synbiotics delivered *in ovo* did not increase FCE with consistent high final body weight. The absence of major effects of the treatment on performance traits can be explained by the fact that the current experiment was conducted with males originating from a highly selected broiler line. These chickens had been genetically previously selected for top performance in the field trials. The goal of synbiotic injection *in ovo* is to maintain the health of the organism, rather than vastly increase its performance. Thus, we achieved numerically improved survivability of the chickens, in both experimental groups (S1, S2).

To date, only a few studies on performance and immunomodulatory effects of synbiotics administered *in ovo* have been conducted [19,20]. Most of the studies on synbiotic administration examined the classical route of delivery, in-feed or in-water. Mookiah *et al*. [44] showed that in-feed supplementation of broiler diet with isomalto-oligosaccharides prebiotic, commercial lactobacilli probiotic, or their combination as a synbiotic resulted in significant improvement of BWG, FI, and FCE. Ghasemi *et al*. [45] observed a similar effect of improved FCE after administration of synbiotics in broiler chicken diets. In contrast, Murshed and Abudabos [46] reported poorer FCE in chickens fed diets with a synbiotic consisting of MOS, beta-glucans, and a highly active strain of *Bacillus subtilis* (DM17299). Moreover, high doses of dietary probiotic tended to increase FCE in later stages of broiler rearing [47]. Mousavi et al. reported that in-feed supplementation of broiler chickens with a synbiotic (*Enterococcus faecium* and oligosaccharides) improved FCE in the starting and growing phase, but not in finishing phase [48]. It might be concluded that despite of the mode of delivery in-feed, in-water, or *in ovo*, the observed effects of synbiotics differ because of biological properties of the bioactive compounds used.

### Microbial community analysis by fluorescent in situ hybridization (FISH)

It is well documented that all segments of the poultry GIT are colonized by different populations of microbiota [49,50]. For instance, ceca are the main fermentative chambers with the highest activity and density of strict anaerobes. For increased performance of broiler chickens, microbiota density and activity (i.e., fermentation) should be minimized in the upper parts of the GIT (i.e., ileum) and increased in the lower GIT segments (i.e., ceca). In the current study, *in ovo* application of S1 and S2 synbiotics triggered different microbial colonization of the ileum and ceca and S1 appeared more effective in modulating a beneficial shift in the GIT microbiota. Supplementation with S1 reduced total microbiota content in the ileum, including the *Bacteroides-*Prevotella, *Clostridium leptum*, the *Eubacterium rectale* cluster, as well as *Lactobacillus* spp.*/Enterococcus* spp. The same treatment increased the *Clostridium leptum* subgroup, *Eubacterium rectale* cluster, and *Lactobacillus* spp.*/Enterococcus* spp. in the ceca. The *Clostridium leptum* subgroup includes many species that belong to *Clostridium*, *Eubacterium*, and *Ruminococcus* genera. These are mostly butyrate-producing and fibrolytic species, which have significant effects on intestinal health. Changes in the *Clostridium leptum* subgroup are indicative of the health status of the gut. A lower number of *Clostridium leptum* subgroup in the ileum and increased number in the ceca could also play a beneficial role in GIT microecology because of higher production of short-chain fatty acids in the ceca and lower competition for nutrients with the host in the ileum. For S1, similar effects were also observed in the *Eubacterium rectale* cluster. Species of this genus produce organic acids, including butyrate, acetate, lactate, or formate, but not propionic and succinic acids, as major products of dietary fiber fermentation [51,52]. Phylogenetic analysis based on 16S rRNA sequences confirmed that *E*. *rectale* belongs to the clostridial cluster XIVa, as defined by Collins et al. [53] within the phylum Firmicutes [54,55]. Therefore, similar to the *Clostridium leptum* subgroup, abundance of *E*. *rectale* is a good indicator of the butyrate producing microbiota, which indirectly affects epithelial cell structure and function, particularly in the lower parts of the GIT.

Microbiota belonging to the *Bacteroides-*Prevotella cluster are one the most frequently isolated pathogens from clinical specimens, from almost all anatomic sites [56]. Moreover, it has been demonstrated that the *Bacteroides*-*Prevotella* cluster can be used to detect fecal contamination in water environments [57]. In present study, application of the S1 synbiotic decreased the number of the *Bacteroides-Prevotella* subgroup in both the ileal and cecal digesta, S2 only in ileum.

*Lactobacilli* are one of the most abundant bacteria groups in GIT of broiler chickens. Lu et al. [58] reported that approximately 70% of the sequences in an ileum-derived 16S rRNA gene library is belonged to *Lactobacillus* genus. However in the ceca, inhabited mostly by strict anaerobes, lactobacilli accounted for only 8% of the library sequences [35,58]. In human and animal nutrition, LAB including *Lactobacillus* genus are usually considered beneficial for the host, mainly because they produce lactic and acetic acids, which leads to pH reduction [59,60] and certain bacteriocins, which help fighting pathogenic bacteria [61,62]. On the other hand, there is also an evidence that some small intestinal LAB, e.g. *L*. *salivarius* or *Enterococcus faecium* might have negative effects on broiler performance [63,64] due to deconjugation of bile salts [65]. Therefore, lower counts of *Lactobacillus* sp.*/Enterococcus* spp. determined in the current study may explain that there were no adverse effects of applied synbiotics on broiler chicken performance, particularly FCE.

#### Gene expression

Modulation of the intestinal microflora has indirect impact on immune system. In this study we demonstrated that S1 triggered up-regulation of immune-related genes in spleen and their down-regulation in cecal tonsils. Our previous *in vivo* experiments proved that synbiotics applied *in ovo* significantly regulated immune-related genes (*IL-1β*, *IL-12p40*, *IFN-γ* and *IL-18*) in spleen and cecal tonsils of adult broiler chickens [19,20]. It was shown that the level of gene expression is time- and tissue-dependent [19,20]. Slawinska et al. determined similar tendency of up-regulation of the immune-related gene expression in spleen followed by clear down-regulation in cecal tonsils after *in ovo* injection of synbiotics [19]. This opposite tendency might be related to different level of exposure both tissues to luminal antigens [20]. Cecal tonsils are part of GALT, thus they are in the close proximity to the intestinal microflora and continuously exposed to microbe-associated molecular patterns (MAMPs). GALT is specialized in anti-inflammatory mechanisms used to eliminate or tolerate microbiota [66]. This mechanism controls the responses of host and develops tolerance towards pathogens, which leads to recognition of commensal bacteria and activation transient and non-inflammatory immune response [67]. *In ovo* administration of synbiotics ensures an early contact between GALT and beneficial bacteria, which promotes development of tolerance mechanisms [19]. This phenomenon explains down-regulation of the immune-related genes in cecal tonsils, that we determined in this study.

S1 and S2 synbiotics delivered *in ovo* exerted a different gene expression pattern of immune-related genes. This difference might be explained by the various composition of both bioactive compounds (S1 and S2). Prebiotic compound in S1 was defined as synergistic to probiotic, while S2 was defined as synergistic to the host. The other explanation could be as follows: bioactive compounds (e.g. prebiotics or probiotics) differ in their affinity to membrane receptors, and as such, they trigger different level of downstream responses in the host cells. In this study, S1 (containing *L*. *salivarius* IBB3154 and GOS) and S2 (containing *L*. *plantarum* IBB3036 and RFO) expressed different immunomodulatory effects in chickens as measured by mRNA level of immune-related genes. To assess the specific properties of lactobacilli to stimulate immune responses, an *in vitro* test had been carried out using chicken macrophage-like HD11 cell line. It confirmed higher stimulatory potential of *Lb*. *salivarius* IBB3154 (~3000 of DEG) vs. *L*. *plantarum* IBB3036 (~500 DEG) in activating gene expression upon direct interaction between macrophages and bacteria (A. Slawinska, personal communication). Synergistic effects of different combinations of prebiotics and probiotics were also evaluated *in vitro* using DT40 cell line. Results from this study indicated that the same bacteria strain combined with a different prebiotic triggered various levels of immune-related gene expression in the host cells [68]. This confirms, that not only a more potent probiotic is needed to express a proper immunomodulatory effect on the host, but it also should be combined with prebiotic.

### Conclusions

The expected role of microflora-stimulating bioactive compounds, such as prebiotics, probiotics or synbiotics is to modulate beneficial changes in intestinal microbiota of the host. In poultry, those bioactive compounds can be supplemented in-feed/in-water or injected *in ovo*. Biological effects of synbiotics, irrespective of the route of delivery, depend solely on careful selection of bioactive compounds (prebiotic and probiotic). In this study, we presented a workflow associated with *in vitro* selection of the synbiotics and its consequences for the downstream animal study, including abundance of intestinal microbial communities, performance parameters, and molecular responses of the immune system. Synbiotic selection *in vitro* provides some indication of their biological potential *in vivo*. Both synbiotics had beneficial effects on the overall status of the organisms defined by low mortality and high production parameters. However, we showed that synbiotic composition affected gene expression in cecal tonsils and the spleen, as well as microflora of the GIT. Out of the two synbiotics tested in this experiment, S1 composed of *L*. *salivarius* and GOS appeared to be more potent in establishing a down-regulatory pattern in the immune-related gene expression in GALT and a beneficial shift in the microbiota composition in the GIT.

## Supporting Information

S1 File Tab A.Tested levels of probiotics and prebiotics for *in ovo* application as a synbiotic on day 12 of incubation. Tab B. Probes used for determination of intestinal microbiota by *in situ* fluorescent hybridization (FISH) Fig A. Fluorescent *in situ* hybridisation (FISH) of single bacterial cells protocol Tab C. Primer sequences used in the RT-qPCR reaction. Tab D. Chicks performance in response to different synbiotics delivered *in ovo*.
(DOCX)

## References

[1] Patterson JA, Burkholder KM. Application of prebiotics and probiotics in poultry production. Poult Sci. 2003;82: 627–631. 12710484 10.1093/ps/82.4.627

[2] Hajati H, Rezaei M. The application of prebiotics in poultry production. Int J Poult Sci. 2010;9: 298–304.

[3] Yang Y, Iji PA, Choct M. Dietary modulation of gut microflora in broiler chickens: a review of the role of six kinds of alternatives to in-feed antibiotics. World Poultry Sci J. 2009;65: 97.

[4] De Vrese M, Schrezenmeir J. Probiotics, prebiotics, and synbiotics. Adv Biochem Eng Biotechnol. 2008;111: 1–66. 10.1007/10_2008_097 18461293

[5] Schrezenmeir J, de Vrese M. Probiotics, prebiotics, and synbiotics—approaching a definition. Am J Clin Nutr. 2001;73: 361–364S. 10.1093/ajcn/73.2.361s 11157342

[6] Roberfroid M. Prebiotics: the concept revisited. J Nutr. 2007;137: 830S–837S. 17311983 10.1093/jn/137.3.830S

[7] Gibson GR, Roberfroid MB. Dietary modulation of the human colonic microbiota: introducing the concept of prebiotics. J Nutr. 1995;125: 1401–1412. 7782892 10.1093/jn/125.6.1401

[8] Naidu AS, Bidlack WR, Clemens RA. Probiotic spectra of lactic acid bacteria (LAB). Crit Rev Food Sci Nutr. 1999;39: 13–126. 10.1080/10408699991279187 10028126

[9] Gilliland SE, Speck ML, Morgan CG. Detection of *Lactobacillus acidophilus* in feces of humans, pigs, and chickens. Appl Microbiol. 1975;30: 541–5. 811162 10.1128/am.30.4.541-545.1975 PMC187227

[10] Sornplang P, Leelavatcharamas V, Soikum C. Heterophil Phagocytic Activity Stimulated by *Lactobacillus salivarius* L61 and L55 Supplementation in Broilers with *Salmonella* Infection. Asian-Australas J Anim Sci. 2015;28: 1657–1661. 10.5713/ajas.15.0359 26580288 PMC4647107

[11] Bodera P. Influence of prebiotics on the human immune system (GALT). Recent Pat Inflamm Allergy Drug Discov. 2008;2: 149–153. 19076004 10.2174/187221308784543656

[12] Pilarski R, Bednarczyk M, Lisowski M, Rutkowski A, Bernacki Z, Wardeńska M, et al Assessment of the effect of alpha-galactosides injected during embryogenesis on selected chicken traits. Folia Biol (Krakow). 2005;53: 13–20. 16212103 10.3409/1734916054663474

[13] Madej JP, Bednarczyk M. Effect of in ovo-delivered prebiotics and synbiotics on the morphology and specific immune cell composition in the gut-associated lymphoid tissue. Poult Sci. 2015;95: 19–29. 10.3382/ps/pev291 26527705

[14] Awad WA, Ghareeb K, Abdel-Raheem S, Böhm J. Effects of dietary inclusion of probiotic and synbiotic on growth performance, organ weights, and intestinal histomorphology of broiler chickens. Poult Sci. 2009;88: 49–56. 10.3382/ps.2008-00244 19096056

[15] Alloui MN, Szczurek W, Science F. The usefulness of prebiotics and probiotics in modern poultry nutrition: a review. Ann Anim Sci. 2013;13: 17–32.

[16] Sansonetti PJ, Di Santo JP. Debugging how bacteria manipulate the immune response. Immunity. 2007;26: 149–61. 10.1016/j.immuni.2007.02.004 17307704

[17] Bar-Shira E, Sklan D, Friedman A. Establishment of immune competence in the avian GALT during the immediate post-hatch period. Dev Comp Immunol. 2003;27: 147–57. 12543128 10.1016/s0145-305x(02)00076-9

[18] Sławińska A, Siwek M, Zylińska J, Bardowski J, Brzezińska J, Gulewicz KA, et al Influence of synbiotics delivered in ovo on immune organs development and structure. Folia Biol (Krakow). 2014;62: 277–85. 25403081 10.3409/fb62_3.277

[19] Slawinska A, Siwek MZ, Bednarczyk MF. Effects of synbiotics injected in ovo on regulation of immune-related gene expression in adult chickens. Am J Vet Res. 2014;75: 997–1003. 10.2460/ajvr.75.11.997 25350090

[20] Płowiec A, Sławińska A, Siwek MZ, Bednarczyk MF. Effect of in ovo administration of inulin and *Lactococcus lactis* on immune-related gene expression in broiler chickens. Am J Vet Res. 2015;76: 975–82. 10.2460/ajvr.76.11.975 26512543

[21] Madej JP, Stefaniak T, Bednarczyk M. Effect of in ovo-delivered prebiotics and synbiotics on lymphoid-organs’ morphology in chickens. Poult Sci. 2015;94: 1209–19. 10.3382/ps/pev076 25877410

[22] Bednarczyk M, Urbanowski M, Gulewicz P, Kasperczyk K, Maiorano G, Szwaczkowski T. Field and *in vitro* Study on Prebiotic Effect of Raffinose Family Oligosaccharides in Chickens. Bull Vet Inst Pulawy. 2011;55: 465–469.

[23] Bogucka J, Dankowiakowska A, Elminowska—Wenda G, Sobolewska A, Szczerba A, Bednarczyk M. Effects of prebiotics and synbiotics delivered in ovo on broiler small intestine histomorphology during the first days after hatching. Folia Biologica (Krakow). 2016;64: 131–134. 10.3409/fb64_3.131 29847074

[24] Miśta D, Króliczewska B, Pecka-Kiełb E, Kapuśniak V, Zawadzki W, Graczyk S, et al Effect of *in ovo* injected prebiotics and synbiotics on the caecal fermentation and intestinal morphology of broiler chicken. Anim Prod Sci. 2016; in press.

[25] Maiorano G, Sobolewska A, Cianciullo D, Walasik K, Elminowska-Wenda G, Slawinska A, et al Influence of *in ovo* prebiotic and synbiotic administration on meat quality of broiler chickens. Poult Sci. 2012;91: 2963–2969. 10.3382/ps.2012-02208 23091157

[26] Pruszynska-Oszmalek E, Kolodziejski PA, Stadnicka K, Sassek M, Chalupka D, Kuston B, et al *In ovo* injection of prebiotics and synbiotics affects the digestive potency of the pancreas in growing chickens. Poult Sci. 2015;94: 1909–16. 10.3382/ps/pev162 26112038

[27] Koenen ME, Van Der Hulst R, Leering M, Jeurissen SHM, Boersma WJA. Development and validation of a new *in vitro* assay for selection of probiotic bacteria that express immune-stimulating properties in chickens in vivo. FEMS Immunol Med Microbiol. 2004;40: 119–127. 10.1016/S0928-8244(03)00306-7 14987730

[28] Gulewicz P, Ciesiołka D, Frias J, Vidal-Valverde C, Frejnagel S, Trojanowska K, et al Simple method of isolation and purification of alpha-galactosides from legumes. J Agric Food Chem. 2000;48: 3120–3123. 10956079 10.1021/jf000210v

[29] de Man JC, Rogosa M, Sharpe ME. A Medium for the Cultivation of Lactobacilli. J Appl Bacteriol. 1960;23: 130–135.

[30] Chlebowska-Śmigiel A, Gniewocz M. Próba zastosowania pullulanu jako stymulatora wzrostu wybranych bakterii probiotycznych i potencjalnie probiotycznych. ŻYWNOŚĆ Nauk Technol Jakość. 2013;3: 111–124.

[31] Gulewicz P, Szymaniec S, Bubak B, Frias J, Vidal-Valverde C, Trojanowska K, et al Biological activity of alpha-galactoside preparations from *Lupinus angustifolius* L. and *Pisum sativum* L. seeds. J Agric Food Chem. 2002;50: 384–389. 11782212 10.1021/jf010973y

[32] Salih NKM, Hutari A, Gaseem WS, Yusoff WMW. Maximization of Growth and Storage of Locally Isolated Lactobacillus salivarius subsp. salivarius with High Stability and Functionality. 25th Southern Biomedical Engineering Conference 2009, 15–17 May 2009, Miami, Florida, USA. IFMBE Proceedings 24: 175–178.

[33] Ayed L, Hamdi M. Culture conditions of tannase production by Lactobacillus plantarum. Biotechnol Lett. 2002;24: 1763–1765.

[34] Rawski M, Kierończyk B, Długosz J, Świątkiewicz S, Józefiak D. Dietary Probiotics Affect Gastrointestinal Microbiota, Histological Structure and Shell Mineralization in Turtles. PLoS One. 2016;11: e0147859 10.1371/journal.pone.0147859 26828367 PMC4735124

[35] Ptak A, Bedford MR, Świątkiewicz S, Żyła K, Józefiak D. Phytase modulates ileal microbiota and enhances growth performance of the broiler chickens. PLoS One. 2015;10: e0119770 10.1371/journal.pone.0119770 25781608 PMC4363628

[36] Ye J, Coulouris G, Zaretskaya I, Cutcutache I, Rozen S, Madden TL. Primer-BLAST: a tool to design target-specific primers for polymerase chain reaction. BMC Bioinformatics. 2012;13: 134 10.1186/1471-2105-13-134 22708584 PMC3412702

[37] Livak KJ, Schmittgen TD. Analysis of relative gene expression data using real-time quantitative PCR and the 2(-Delta Delta C(T)) Method. Methods. 2001;25: 402–408. 10.1006/meth.2001.1262 11846609

[38] Goderska K, Nowak J, Czarnecki Z. Comparison of the growth of *Lactobacillus acidophilus* and *Bifidobacterium bifidum* species in media supplemented with selected saccharides including prebiotics. Acta Sci Pol Technol Aliment. 2008;7: 5–20.

[39] Kneifel W, Rajal A, Kulbe KD. *In vitro* growth behaviour of probiotic bacteria in culture media with carbohydrates of prebiotic importance. Microb Ecol Health Dis. 2000;12: 27–34.

[40] Cummings JH, Macfarlane GT, Englyst HN. Prebiotic digestion and fermentation. Am J Clin Nutr. 2001;73: 415–420. 10.1093/ajcn/73.2.415s 11157351

[41] Fuller R. Probiotics in man and animals. J Appl Bacteriol. 1989;66: 365–378. 2666378

[42] Bednarczyk M, Stadnicka K, Kozłowska I, Abiuso C, Tavaniello S, Dankowiakowska A, et al Influence of different prebiotics and mode of their administration on broiler chicken performance. Animal. 2016; 1271–9. 10.1017/S1751731116000173 26936310

[43] Furuse M, Yokota H. Effect of the gut microflora on chick growth and utilisation of protein and energy at different concentrations of dietary protein. Br Poult Sci. 1985;26: 97–104. 10.1080/00071668508416791 3971197

[44] Mookiah S, Sieo CC, Ramasamy K, Abdullah N, Ho YW. Effects of dietary prebiotics, probiotic and synbiotics on performance, caecal bacterial populations and caecal fermentation concentrations of broiler chickens. J Sci Food Agric. 2014;94: 341–348. 10.1002/jsfa.6365 24037967

[45] Ghasemi HA, Shivazad M, Mirzapour Rezaei SS, Torshizi MAK. Effect of synbiotic supplementation and dietary fat sources on broiler performance, serum lipids, muscle fatty acid profile and meat quality. Br Poult Sci. 2015; 10.1080/00071668.2015.1098766 26654967

[46] Murshed M, Abudabos A. Effects of the Dietary Inclusion of a Probiotic, a Prebiotic or their Combinations on the Growth Performance of Broiler Chickens. Rev Bras Ciência Avícola. 2015;17: 99–103.

[47] Mountzouris KC, Tsitrsikos P, Palamidi I, Arvaniti A, Mohnl M, Schatzmayr G, et al Effects of probiotic inclusion levels in broiler nutrition on growth performance, nutrient digestibility, plasma immunoglobulins, and cecal microflora composition. Poult Sci. 2010;89: 58–67. 10.3382/ps.2009-00308 20008803

[48] Mousavi SMAA, Seidavi A, Dadashbeiki M, Kilonzo-Nthenge A, Nahashon SN, Laudadio V, et al Effect of a synbiotic (Biomin®IMBO) on growth performance traits of broiler chickens. Europ Poult Sci 2015;79.

[49] Salanitro JP, Blake IG, Muirehead PA, Maglio M, Goodman JR. Bacteria isolated from the duodenum, ileum, and cecum of young chicks. Appl Environ Microbiol. 1978;35: 782–90. 646359 10.1128/aem.35.4.782-790.1978 PMC242923

[50] Pan D, Yu Z. Intestinal microbiome of poultry and its interaction with host and diet. Gut Microbes. 2014;5: 108–19. 10.4161/gmic.26945 24256702 PMC4049927

[51] Krumholz LR, Bryant MP. *Syntrophococcus sucromutans* sp. nov. gen. nov. uses carbohydrates as electron donors and formate, methoxymonobenzenoids or *Methanobrevibacter* as electron acceptor systems. Arch Microbiol. 1986;143: 313–318.

[52] Andreesen JR. The genus *Eubacterium* In: Balows A, Trüper HG, Dworkin M, Harder W, Schleifer KH, editors. The Prokaryotes. 2nd edit New York: Springer Verlag; 1992 pp. 1914–1924.

[53] Collins MD, Lawson PA, Willems A, Cordoba JJ, Fernandez-Garayzabal J, Garcia P, et al The phylogeny of the genus *Clostridium*: proposal of five new genera and eleven new species combinations. Int J Syst Bacteriol. 1994;44: 812–826. 10.1099/00207713-44-4-812 7981107

[54] Barcenilla A, Pryde SE, Martin JC, Duncan SH, Stewart CS, Henderson C, et al Phylogenetic Relationships of Butyrate-Producing Bacteria from the Human Gut. Appl Environ Microbiol. 2000;66: 1654–1661. 10742256 10.1128/aem.66.4.1654-1661.2000 PMC92037

[55] Aminov RI, Walker AW, Duncan SH, Harmsen HJM, Welling GW, Flint HJ. Molecular diversity, cultivation, and improved detection by fluorescent in situ hybridization of a dominant group of human gut bacteria related to *Roseburia* spp. or *Eubacterium rectale*. Appl Environ Microbiol. 2006;72: 6371–6376. 10.1128/AEM.00701-06 16957265 PMC1563657

[56] Falagas ME, Siakavellas E. *Bacteroides*, *Prevotella*, and *Porphyromonas* species: a review of antibiotic resistance and therapeutic options. Int J Antimicrob Agents. 2000;15: 1–9. 10856670 10.1016/s0924-8579(99)00164-8

[57] Kobayashi A, Sano D, Hatori J, Ishii S, Okabe S. Chicken- and duck-associated *Bacteroides-Prevotella* genetic markers for detecting fecal contamination in environmental water. Appl Microbiol Biotechnol. 2013;97: 7427–7437. 10.1007/s00253-012-4469-2 23053113

[58] Lu J, Idris U, Harmon B, Hofacre C, Maurer JJ, Lee MD. Diversity and Succession of the Intestinal Bacterial Community of the Maturing Broiler Chicken. Appl Environ Microbiol. 2003;69: 6816–6824. 10.1128/AEM.69.11.6816-6824.2003 14602645 PMC262306

[59] Engberg RM, Hedemann MS, Steenfeldt S, Jensen BB. Influence of whole wheat and xylanase on broiler performance and microbial composition and activity in the digestive tract. Poult Sci. 2004;83: 925–938. 15206619 10.1093/ps/83.6.925

[60] Józefiak D, Sip A, Rutkowski A, Rawski M, Kaczmarek S, Wołuń-Cholewa M, et al Lyophilized *Carnobacterium divergens* AS7 bacteriocin preparation improves performance of broiler chickens challenged with *Clostridium perfringens*. Poult Sci. 2012;91: 1899–1907. 10.3382/ps.2012-02151 22802184

[61] Nazef L, Belguesmia Y, Tani A, Prévost H, Drider D. Identification of lactic acid bacteria from poultry feces: evidence on anti-campylobacter and anti-listeria activities. Poult Sci. 2008;87: 329–334. 10.3382/ps.2007-00282 18212377

[62] Józefiak D, Sip A. Bacteriocins In Poultry Nutrition–A Review / Bakteriocyny w żywieniu drobiu–artykuł przeglądowy. Ann Anim Sci. 2013;13: 449–462.

[63] Engberg RM, Hedemann MS, Leser TD, Jensen BB. Effect of zinc bacitracin and salinomycin on intestinal microflora and performance of broilers. Poult Sci. 2000;79: 1311–1319. 11020077 10.1093/ps/79.9.1311

[64] Guban J, Korver DR, Allison GE, Tannock GW. Relationship of Dietary Antimicrobial Drug Administration with Broiler Performance, Decreased Population Levels of *Lactobacillus salivarius*, and Reduced Bile Salt Deconjugation in the Ileum of Broiler Chickens. Poult Sci. 2006;85: 2186–2194. 17135676 10.1093/ps/85.12.2186

[65] Knarreborg A, Engberg RM, Jensen SK, Jensen BB. Quantitative determination of bile salt hydrolase activity in bacteria isolated from the small intestine of chickens. Appl Environ Microbiol. 2002;68: 6425–6428. 10.1128/AEM.68.12.6425-6428.2002 12450872 PMC134412

[66] Brisbin JT, Gong J, Sharif S. Interactions between commensal bacteria and the gut-associated immune system of the chicken. Anim Health Res Rev. 2008;9: 101–110. 10.1017/S146625230800145X 18541076

[67] Galdeano CM, de Moreno de LeBlanc A, Vinderola G, Bonet MEB, Perdigón G. Proposed model: mechanisms of immunomodulation induced by probiotic bacteria. Clin Vaccine Immunol. 2007;14: 485–92. 10.1128/CVI.00406-06 17360855 PMC1865623

[68] Sławińska A, Siwek M, Bednarczyk M. In vitro screening of immunomodulatory properties of synbiotics in chicken DT40 cell line. Anim Sci Pap Rep. 2016;34: 81–93.

[69] Franks AH, Harmsen HJM, Raangs GC, Jansen GJ, Schut F, Welling GW. Variations of bacterial populations in human feces quantified by fluorescent in situ hybridization with group-specific 16S rRNA-targeted oligonucleotide probes. Appl Environ Microbiol 1998; 64: 3336–3345. 9726880 10.1128/aem.64.9.3336-3345.1998 PMC106730

[70] Sghir A, Antonopoulos D, Mackie RI. Design and evaluation of a *Lactobacillus* group-specific ribosomal RNA-targeted hybridization probe and its application to the study of intestinal microecology in pigs. Syst Appl Microbiol. 1998;21: 291–296. 10.1016/S0723-2020(98)80036-2 9704114

[71] Harmsen HJM, Elfferich P, Schut F, Welling GW. A 16S rRNA-targeted probe for detection of lactobacilli and enterococci in faecal samples by fluorescent in situ hybridization. Microb Ecol Health Dis. 1999;11: 3–12.

[72] Manz W, Szewzyk U, Ericsson P, Amann RI, Schleifer KH, Stenström T-A. In-situ identification of bacteria in drinking water and adjoining biofilms by hybridization with 16S ribosomal RNA-directed and 23S ribosomal RNA-directed fluorescent oligonucleotide probes. Appl Environ Microbiol. 1993;59: 2293–2298. 8357261 10.1128/aem.59.7.2293-2298.1993 PMC182271

[73] Chiang HI, Berghman LR, Zhou H. Inhibition of NF-kB 1 (NF-kBp50) by RNA interference in chicken macrophage HD11 cell line challenged with *Salmonella* enteritidis. Genet Mol Biol. 2009;32: 507–515. 10.1590/S1415-47572009000300013 21637513 PMC3036038

[74] Brisbin JT, Gong J, Parvizi P, Sharif S. Effects of lactobacilli on cytokine expression by chicken spleen and cecal tonsil cells. Clin Vaccine Immunol. 2010;17: 1337–1343. 10.1128/CVI.00143-10 20668142 PMC2944448

[75] De Boever S, Vangestel C, De Backer P, Croubels S, Sys SU. Identification and validation of housekeeping genes as internal control for gene expression in an intravenous LPS inflammation model in chickens. Vet Immunol Immunopathol. 2008;122: 312–317. 10.1016/j.vetimm.2007.12.002 18272235

[76] Sevane N, Bialade F, Velasco S, Rebolé A, Rodríguez ML, Ortiz LT, et al Dietary inulin supplementation modifies significantly the liver transcriptomic profile of broiler chickens. PLoS One. 2014;9. 10.1371/journal.pone.0098942 PMC4051581 24915441

