# Supplementary material for: Synbiotics for Broiler Chickens—In Vitro Design and Evaluation of the Influence on Host and Selected Microbiota Populations following In Ovo Delivery
Source: PLoS One. 2017 Jan 3;12(1):e0168587. doi: 10.1371/journal.pone.0168587 (PMC5207659; doi:10.1371/journal.pone.0168587)
Supplement: S1 File — Tab A.Tested levels of probiotics and prebiotics for in ovo application as a synbiotic on day 12 of incubation. Tab B. Probes used for determination of intestinal microbiota by in situ fluorescent hybridization (FISH) Fig A. Fluorescent in situ hybridisation (FISH) of single bacterial cells protocol Tab C. Primer sequences used in the RT-qPCR reaction. Tab D. Chicks performance in response to different synbiotics delivered in ovo. (DOCX) [file pone.0168587.s001.docx]

**Tab A. Tested levels of probiotics and prebiotics for *in ovo* application as a synbiotic on day 12 of incubation.**

| **Control**  0.2 mM physiological saline (0.9 %) | **bacteria (cfu/egg)** | **prebiotic (mg/egg)** |
| --- | --- | --- |
|  | 0.00 | 0.00 |
| **Synbiotic 1**  *Lactobacillus salivarius* IBB3154  + GOS | 10^3^  10^4^  10^5^  10^3^  10^4^  10^5^ | 2.0  5.0 |
| **Synbiotic 2**  *Lactobacillus plantarum* IBB 3036  + RFO | 10^3^  10^4^  10^5^  10^3^  10^4^  10^5^ | 2.0  5.0 |

**Tab B. Probes used for determination of intestinal microbiota by *in situ* fluorescent hybridization (FISH)**

| **Target** | **Probe** | | **Sequence (5' to 3')** | **Reference** |
| --- | --- | --- | --- | --- |
| *Clostridium coccoides*—*Eubacterium rectale* cluster | | Erec482 | GCTTCTTAGTCARGTACCG | 69 |
| *Clostridium leptum* subgroup | | Clept1240 | GTTTTRTCAACGGCAGTC | 70 |
| *Lactobacillus spp./ Enterococcus spp.* | | Lab158 | GGTATTAGCAYCTGTTTCCA | 71 |
| *Bacteroides*-Prevotella cluster | | Bac303 | CCAATGTGGGGGACCTT | 72 |

**Fig A. Fluorescent *In situ* Hybridisation (FISH) of single bacterial cells protocol**


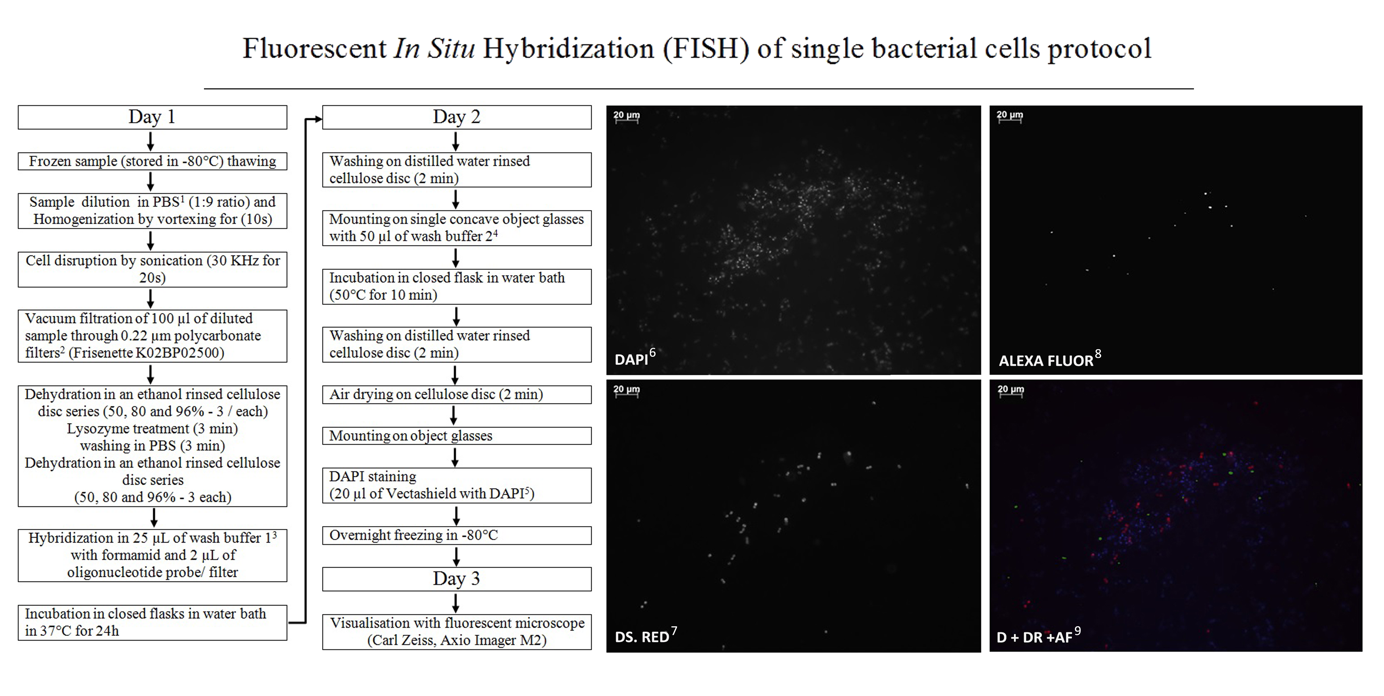


**Tab C. Primer sequences used in the RT-qPCR reaction.**

| Gene | NCBI gene ID | Primer sequences  (5’ 🡪 3’) | Annealing temp. (°C) | Reference |
| --- | --- | --- | --- | --- |
| IL4 | 416330 | F: GCTCTCAGTGCCGCTGATG  R: GGAAACCTCTCCCTGGATGTC | 58 | 19 |
| IL6 | 395337 | F: AGGACGAGATGTGCAAGAAGTTC  R: TTGGGCAGGTTGAGGTTGTT | 58 | 73 |
| IL12p40 | 404671 | F: TTGCCGAAGAGCACCAGCCG  R: CGGTGTGCTCCAGGTCTTGGG | 65 | 74 |
| IL8 | 396495 | F: AAGGATGGAAGAGAGGTGTGCTT  R: GCTGAGCCTTGGCCATAAGT | 58 | 19 |
| IL18 | 395312 | F: GAAACGTCAATAGCCAGTTGC  R: TCCCATGCTCTTTCTCACAACA | 58 | 74 |
| IL1β | 395196 | F: GGAGGTTTTTGAGCCCGTC  R: TCGAAGATGTCGAAGGACTG | 58 | this study |
| IFNγ | 396054 | F: ACACTGACAAGTCAAAGCCGC  R: AGTCGTTCATCGGGAGCTTG | 58 | 74 |
| IFNβ | 554219 | F: ACCAGATCCAGCATTACATCCA  R: CGCGTGCCTTGGTTTACG | 58 | 19 |
| UB | 396425 | F: GGGATGCAGATCTTCGTGAAA  R: CTTGCCAGCAAAGATCAACCTT | 58 | 75 |
| G6PD | 428188 | F: CGGGAACCAAATGCACTTCGT  R: GGCTGCCGTAGAGGTATGGGA | 58 | 76 |

| **Item** | **Body Weight Gain (g)** | | | | | | | | | **Feed Intake (g)** | | | | | | | |
| --- | --- | --- | --- | --- | --- | --- | --- | --- | --- | --- | --- | --- | --- | --- | --- | --- | --- |
|  | **1-10 day** | | | | **10-20 day** | | **20-41 day** | | **1-41 day** | **1-10 day** | | **10-20 day** | | **20-41 day** | | **1-41 day** | |
| **Control** | | 205 | 615 | | | 2286 | | 3105 | | 254^a^ | 862 | | 3824 | | 4940 | |  |
| **S1** | | 204 | 592 | | | 2291 | | 3087 | | 247^b^ | 839 | | 3844 | | 4930 | |  |
| **S2** | | 201 | 614 | | | 2255 | | 3070 | | 258^a^ | 857 | | 3783 | | 4898 | |  |
| **SEM** | | 2.1876 | 11.2619 | | | 14.4793 | | 9.7998 | | 1.3291 | 11.0976 | | 19.6773 | | 24.4097 | |  |
| **P-value** | | P>0.05 | P>0.05 | | | P>0.05 | | P>0.05 | | P<0.05 | P>0.05 | | P>0.05 | | P>0.05 | |  |
| **Item** | | **Feed Conversion Efficiency (g/g)** | | | | | | | | **Mortality (%)** | | | | | | |  |
|  | | **1-10 day** | | **10-20 day** | | **20-41 day** | | **1-41 day** | |  |  |  |  |  |  |  |  |
| **Control** | | 1.24 | | 1.41 | | 1.67 | | 1.59 | | 1.83 | | | | | | |  |
| **S1** | | 1.21 | | 1.42 | | 1.68 | | 1.6 | | 0.83 | | | | | | |  |
| **S2** | | 1.29 | | 1.4 | | 1.68 | | 1.6 | | 1.17 | | | | | | |  |
| **SEM** | | 0.0146 | | 0.0087 | | 0.0121 | | 0.0067 | | 0.2473 | | | | | | |  |
| **P-value** | | P>0.05 | | P>0.05 | | P>0.05 | | P>0.05 | | P>0.05 | | | | | | |  |

**Tab D. Chicks performance in response to different synbiotics delivered *in ovo*.**

(S1) *L. salivarius* + GOS, (S2) *L. plantarum* +RFO.

Eggs were injected *in ovo* with 0.2 mL of either S1 or S2 aqueous solution on day 12. of egg incubation. Control eggs were injected with 0.2 mL of physiological saline. Each experimental group was represented by 600 roosters which were split into 8 pens (75 individuals/pen). Each pen was considered as a replicate. Roosters were reared for 42 days. Non-sexed chickens (n=8000, originating from the same hatching group) were grown beside the experimental chickens to fill the space of the chicken house. The data was analyzed using General Linear Models procedure. Duncan post hoc test was applied to compare the mean values among the experimental groups (a,b: P<0.05).
